# State of the art CRISPR-based strategies for cancer diagnostics and treatment

**DOI:** 10.1186/s40364-024-00701-x

**Published:** 2024-12-18

**Authors:** Emma Di Carlo, Carlo Sorrentino

**Affiliations:** 1 Department of Medicine and Sciences of Aging, ”G. d’Annunzio University” of Chieti- Pescara, Via dei Vestini, Chieti, 66100 Italy; 2 Anatomic Pathology and Immuno-Oncology Unit, Center for Advanced Studies and Technology (CAST), ”G. d’Annunzio” University of Chieti-Pescara, Via L. Polacchi 11, Chieti, 66100 Italy

**Keywords:** CRISPR, Cas9, Cas12, Cas13, Personalized anti-cancer therapy, Genome-editing, RNA-targeting, SHERLOCK, DETECTR, Molecular diagnostics

## Abstract

Clustered Regularly Interspaced Short Palindromic Repeats (CRISPR) technology is a groundbreaking and dynamic molecular tool for DNA and RNA “surgery”. CRISPR/Cas9 is the most widely applied system in oncology research. It is a major advancement in genome manipulation due to its precision, efficiency, scalability and versatility compared to previous gene editing methods. It has shown great potential not only in the targeting of oncogenes or genes coding for immune checkpoint molecules, and in engineering T cells, but also in targeting epigenomic disturbances, which contribute to cancer development and progression. It has proven useful for detecting genetic mutations, enabling the large-scale screening of genes involved in tumor onset, progression and drug resistance, and in speeding up the development of highly targeted therapies tailored to the genetic and immunological profiles of the patient’s tumor. Furthermore, the recently discovered Cas12 and Cas13 systems have expanded Cas9-based editing applications, providing new opportunities in the diagnosis and treatment of cancer. In addition to traditional cis-cleavage, they exhibit trans-cleavage activity, which enables their use as sensitive and specific diagnostic tools. Diagnostic platforms like DETECTR, which employs the Cas12 enzyme, that cuts single-stranded DNA reporters, and SHERLOCK, which uses Cas12, or Cas13, that specifically target and cleave single-stranded RNA, can be exploited to speed up and advance oncological diagnostics. Overall, CRISPR platform has the great potential to improve molecular diagnostics and the functionality and safety of engineered cellular medicines. Here, we will emphasize the potentially transformative impact of CRISPR technology in the field of oncology compared to traditional treatments, diagnostic and prognostic approaches, and highlight the opportunities and challenges raised by using the newly introduced CRISPR-based systems for cancer diagnosis and therapy.

## The CRISPR family

The CRISPR (Clustered Regularly Interspaced Short Palindromic Repeats) family refers to a group of DNA sequences found in the genomes of bacteria and archaea. These sequences are part of an adaptive immune system that provides these microorganisms with resistance to foreign genetic elements, such as plasmids and phages [1]. The CRISPR components include: **(a) CRISPR arrays** represented by repeated DNA sequences interspaced with unique sequences called spacers, which are derived from viruses that have previously attacked the bacteria. Each spacer sequence corresponds to a fragment of viral DNA; **(b) Cas (CRISPR-associated) proteins**, a family of proteins that are essential for the CRISPR immune response, which has several members including the well-known Cas9 protein [2] and the most recently investigated Cas12 and Cas13, each with unique functions and properties [3, 4], as summarized in the Tables 1a and 1b.

**Table 1a:** Characteristics of the different CRISPR systems

Type of CRISPR/Cas system	Enzyme	Size	Guide spacer length	Total guide length	PAM	Cut
Type II	Cas9	~ 1,000–1,600 aa	18–24 nt	~ 100 nt (sgRNA)	3-NGG (SpCas9) 3-NNGRRT (SaCas9) 3-NNNNGATT (NmCas9)	Blunt-ended dsDNA break
Type V	Cas12	~ 1,100–1,300 aa	18–25 nt	42–44 nt	5-TTTN	*Subtype V-A*: 5 nt 5 overhang dsDNA break *Subtype V-B*: 5 nt 5 overhang - DdsDNA break
Type VI	Cas13	~ 900—1,300 aa	22–30 nt	52–66 nt	*Subtype VI-A*: 3-H *Subtype VI-B*: 5-D and 3-NAN or NNA *Subtype VI-D*: none	ssRNA

PAM (Protospacer-Adjacent motif): short, conserved sequence on the strand of DNA adjacent to the target site. ssRNA: single stranded RNA

**Table 1b:** Characteristics of the different CRISPR systems

Type of CRISPR/Cas system	Subtypes	Organisms	Mechanism of action	Applications
Type II	*II-A* *II-B* *II-C*	• *Streptococcus pyogenes* • *Streptococcus thermophilus* • *Staphylococcus aureus* • *Neisseria meningitidis* • *Campylobacter jejuni*	Cas9 creates double-stranded DNA breaks, near the PAM site, which can then be repaired by either non-homologous end joining or homologous recombination with a donor template DNA to create site-specific edits. Type II-A Cas9s generally have high genome editing efficiency, but off-target cleavage at unintended genome sites can be a disadvantage.	− Cancer immunotherapy − Genome-wide screening for identification of cancer genes − Gene therapy − Viral diseases diagnosis and treatment − Tissue regeneration
Type V	*V-A (Cpf1)* *V-B (C2c1)*	• *Francisella novicida*, • *Acidaminococcus sp.* • *Lachnospiraceae sp.* • *Prevotella sp.*	Both subtypes generate staggered DNA breaks distal to the PAM site and enable versatile multiple genomic loci targeting by processing its own guide RNAs.	Cas12 has been engineered as a platform for epigenome editing, and it was recently discovered that Cas12 can indiscriminately chop up single-stranded DNA once activated by a target DNA molecule matching its spacer sequence. This property makes Cas12 a powerful tool for: − Multiplex genome editing − Epigenome editing − Diagnostic of viral diseases The subtype V-B has a significantly smaller size (1108 aa) than subtype V-A (1307 aa), making it a more suitable tool for gene therapy applications that use a viral delivery system. Furthermore, it is very sensitive to mismatches between the guide RNA and target DNA, thus causing less off-target effects.
Type VI	*VI-A* *VI-B* *VI-C* *VI-D*	• *Leptotrichia buccalis* • *Leptotrichia shahii* • *Ruminococcus flavefaciens* • *Bergeyella zoohelcum* • *Prevotella buccae* • *Listeria seeligeri*	Cas13 targets RNA, not DNA. Once it is activated by a ssRNA sequence complementary to its crRNA spacer, it destroys all nearby RNAs, regardless of their sequence.	Cas13 is an outlier in the CRISPR world because it targets RNA, not DNA. Its applications include: − Specific RNA knock-down or editing without altering genome sequence − Diagnostic of viral diseases (RNA viruses) Subtype VI-B had consistently increased levels of knock-down relative to subtype VI-A. (average of 92.3% *versus* 40.1%)

crRNA (CRISPR RNA): RNA sequence complementary to the target DNA

Mechanistically, the CRISPR/Cas system operates in three stages: **1.** **Adaptation**. When a bacterium is invaded by a virus, the CRISPR system captures a segment of the viral DNA and inserts it into the CRISPR array as a new spacer [5]. **2.** **Expression**. The CRISPR array is transcribed into a long RNA molecule, which is then processed into shorter CRISPR RNAs (crRNAs) containing individual spacer sequences. **3.** **Interference**, the process by which the CRISPR system inactivates the invading phage. The crRNAs guide the Cas proteins to the complementary viral DNA sequence, once there the Cas protein cleaves the DNA, neutralizing the viral threat [6]. The CRISPR/Cas systems are classified into two main classes and further into types and subtypes [7]. **Class 1** involves multi-protein complexes for interference, e.g. **Type I** (Cas3), **Type III** (Cas10) and **Type IV** (Cas5 and Cas7). **Class 2** uses a single, large Cas protein for interference, e.g. **Type II** (Cas9), **Type V** (Cas12) and **Type VI** (Cas13) [8].

## A brief history of CRISPR-based systems

The CRISPR/Cas9 system is one of the most revolutionary scientific discoveries of recent decades, bringing gene editing to levels of precision and reliability that were unthinkable before [9]. The first CRISPR sequence was identified in 1987 by Ishino, who while analyzing the inhibitor of apoptosis (*iap*) gene in *Escherichia coli*, identified a series of 29-nucleotide repeats, interspersed with 32 to 33 nucleotide variable sequences, at the 3′-end of the gene [10]. In 1993, Mojica revealed the presence of similar DNA sequences in the halophilic archaeon *Haloferax mediterranei* [11] and, in 2000, Jansen coined the term CRISPR, highlighting the distinctive characteristic of this family of DNA sequences, which, unlike any other class of repetitive DNA, are interspaced by similarly sized non-repetitive DNA [12]. However, the biological function of CRISPR remained unknown until 2005, when Mojica demonstrated that these sequences were derived from viruses that had previously infected bacteria, suggesting that they functioned as an immune “memory” [13].

The breakthrough came in 2012, when Doudna and Charpentier demonstrated that the CRISPR/Cas9 system uses a dual-RNA structure, consisting of a crRNA and a trans-activating crRNA (tracrRNA), to guide the action of the Cas9 protein. This dual-RNA structure could be engineered as a single guide RNA (sgRNA) to direct the Cas9 enzyme towards specific regions of genomic DNA, allowing for extremely precise gene editing [14].

The first applications of CRISPR in cancer research was carried out in 2013 by Zhang [15] and Church [16], who demonstrated that, by using multiple gRNA sequences, the Cas9 enzyme can be directed to different sites in human and mouse cell lines, enabling multiple genome editing, ranging from small insertions and deletions (indels) to chromosomal deletions.

In 2015, Makarova defined two classes of CRISPR/Cas systems, with distinct evolutionary histories. The Class 1 system is characterized by multi-subunit effector complexes (type I, III, and IV), while the Class 2 system is associated with single-protein effector modules (types II, V, and VI) [17]. This classification provided a framework for understanding the different molecular architectures of CRISPR/Cas systems, stimulating scientists to search and identify previously unknown subtypes and novel proteins. Between 2015 and 2016, Abudayyeh identified two new CRISPR-based systems, in *Francisella novicida*, the Cas12 protein, a Class 2, Type V enzyme that could introduce targeted double-stranded breaks in DNA and, in *Leptotrichia shahii*, the Cas13 protein, a unique Class 2, Type VI CRISPR-associated enzyme, distinguished by its ability to target RNA rather than DNA [18, 19].

In 2016, with the emergence of immunotherapy, Marson explored the use of CRISPR/Cas9 to modify immune cells to enhance their ability to target cancer, and this approach was essential for the development of chimeric antigens receptor (CAR)-T cell therapies [20]. The CRISPR/Cas9-based editing method allowed the precise integration of the modified T cell receptor (TCR) in primary human T cells, without compromising their function, resulting in highly purified populations that stably expressed the new TCR [20]. In 2017, Patel used the CRISPR system, together with a machine-learning computational model, to identify genes essential for tumor progression in melanoma cell lines, such as collagen type XVII alpha 1 (*COL17A1*), twinfilin-1 (*TWF1*), microRNA 101-2 (*Hsa-Mir-101-2*) and ribosomal protein L23 (*RPL23*) [21]. These large-scale screenings allowed researchers to identify vulnerabilities in cancer cells that could be targeted for therapy. In 2020, Stadtmauer launched the first clinical trial testing the safety and feasibility of CRISPR/Cas9 editing in cancer patients [22]. The trial involved the CRISPR/Cas9-based deletion of the genes encoding TCRα, TCRβ and programmed cell death protein 1 (PD-1; PDCD1), in T cells derived from patients, followed by lentiviral transduction of a transgenic TCR specific for the cancer antigen New York oesophageal squamous cell carcinoma 1 (*NY-ESO-1*). The subsequent re-infusion of the engineered T cells resulted in durable engraftment, with editing in all genomic loci, and the modified cells persisted, in the circulation, for up to 9 months, indicating minimal immunogenicity [22].

Below, light will be shed on the current use and future perspectives offered by Cas9-, Cas12- and Cas13-based methods in experimental and clinical oncology.

## Using the CRISPR/Cas9 system for cancer therapy and diagnosis

**Fig. 1:**
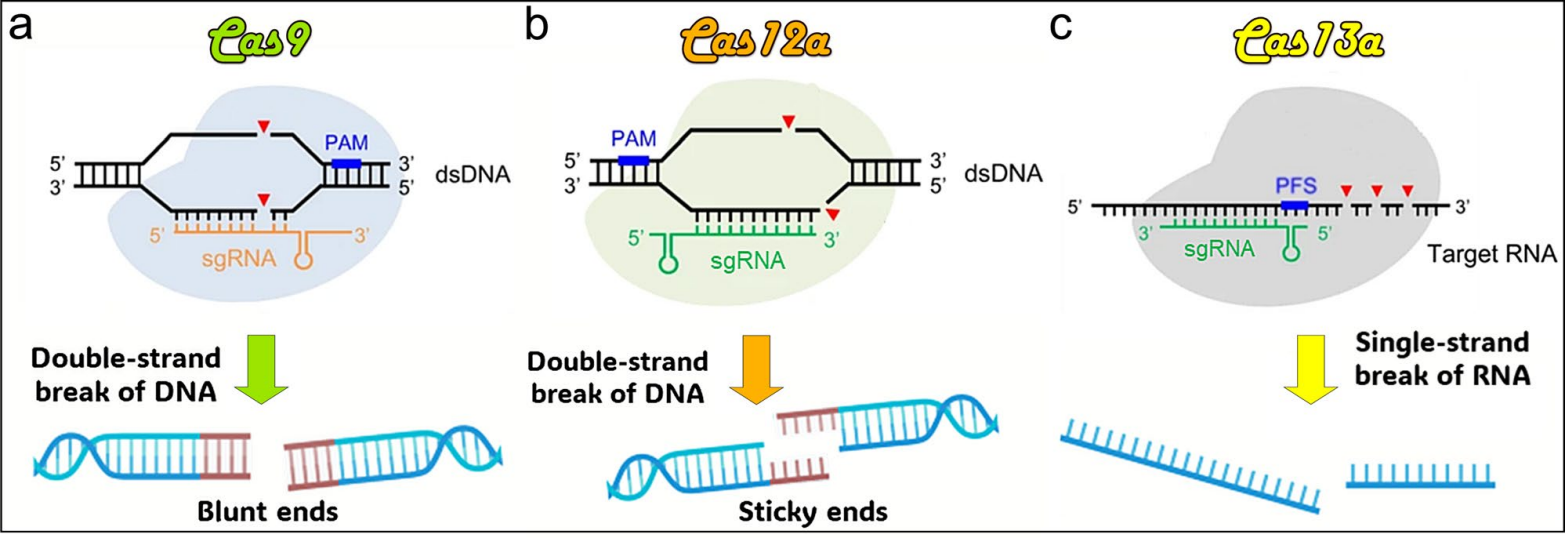
Illustration of DNA strand cleavage tools. **(a)** Cas9 cleaves DNA double strands to form blunt ends. **(b)** Cas12a cuts DNA double strands to form sticky ends. **(c)** Cas13a recognizes and cleaves RNA strands. PAM: protospacer adjacent motif. PFS: protospacer flanking sequence. sgRNA: single guide RNA

Cas9 (Class 2, Type II system) has a cis-cleavage function and causes double-stranded (ds)DNA breaks [23]. Cleavage activity for single-stranded (ss)DNA and ssRNA has also been reported in the presence of Protospacer Adjacent Motif (PAM)-presenting oligonucleotides (PAMmers) sequences designed to complement the target sequence (Fig. 1a) [24]. This property has been successfully used, coupled with real-time fluorescence analysis modalities, for the detection of specific nucleic acid targets [25]. Gene editing with the CRISPR/Cas9 system has shown great potential in both the treatment and diagnosis of cancer.

### Applications of CRISPR/Cas9 for cancer treatment include gene editing and repair, immune enhancement strategies, and removal of anti-cancer drug resistance

1. **Gene editing and repair.** CRISPR/Cas9 can precisely target and edit genes responsible for cancer initiation or progression, potentially deactivating oncogenes or activating tumor suppressor genes [26].

- **Targeting oncogenes**. CRISPR/Cas9 can be used to specifically target, and knock-out, mutated or overexpressed oncogenes, such as *MYC*, *KRAS*, and *EGFR*, which drive tumor growth and progression [27, 28]. Interruption of the constitutively active tyrosine-kinase BCR/ABL1 Fusion Oncogene (FO) led to apoptosis and induced proliferative arrest of primary leukemic stem cells [29]. Disrupting two established FOs, involving either a transcription factor, EWSR1-FLI1 (EF), in Ewing sarcoma, or a tyrosine kinase, BCR-ABL1 (BA), in chronic myeloid leukemia, resulted in efficient cancer cell death and reduced tumor burden and mortality [30]. KRAS is the most frequently mutated oncogene in human cancers, thus representing an important therapeutic target. Knocking out mutated KRAS using CRISPR has been shown to inhibit colorectal cancer cell proliferation, both in vitro and in vivo [27].
  CRISPR/Cas9 as proven to be effective in knocking out novel cancer drivers, such as interleukin(IL)-30 (IL27/p28) [31], which can both drive cancer stem cell expansion and exert immunosuppressive activity in the colorectal, prostate and breast cancer microenvironment [32–34]. Human IL-30 can be found as a membrane-anchored cytokine, expressed by cancer cells and by tumor-infiltrating macrophages and myeloid-derived suppressor cells (MDSCs) [33, 35] and plays a critical role in tumor onset and progression, by triggering a cascade of proinflammatory and oncogenic events, in association with the development of a robust vascular network [36, 37]. CRISPR/Cas9-mediated deletion of IL30 gene in stem or differentiated cancer cells has proven effective in hindering tumor growth and improve survival in colorectal and prostate cancer xenograft models [32, 33]. Moreover, intravenous administration of immunoliposomes that selectively convey Cas9gRNA-hIL30 complex for IL30 genome editing at the tumor, or metastatic sites, has shown to inhibit tumor growth and progression and improve survival without evident toxicity in xenograft and syngeneic PC models [38, 39].

- **Restoring tumor suppressor genes**. CRISPR/Cas9 can be used to repair mutations or deletion in tumor suppressor genes, such as *TP53*, *BRCA1*, *RB1* and *PTEN*, restoring their function and thereby halting disease progression [40, 41]. However, to make CRISPR/Cas9-mediated gene repair a viable therapeutic option, technical challenges, such as the low efficiency of Homology-Directed Repair (HDR), cell cycle dependence and the design of highly specific single guide RNAs (sgRNAs) to minimize off-target effects, urgently need to be addressed.
- **Epigenome editing.** Epigenetic aberrations are known to cooperate with genetic alterations in driving the cancer phenotype [42]. Targeting the cancer epigenome with CRISPR technology is currently being developed for cancer therapy [43, 44]. By fusing a catalytically dead form of Cas9, namely nuclease-deficient Cas9 (dCas9), with epigenetic modifiers, such as Dnmt3a (a DNA methylation enzyme) or Tet1 (a DNA demethylation enzyme), gene expression can be modulated without changing the DNA sequence [45]. These tools can add or remove epigenetic marks and reverse cancer-associated epigenetic changes [45]. Epigenetic therapies have proven to sensitize patients to the reversal of immune tolerance and to improve the efficacy of immune checkpoint therapies [46] in metastatic non-small cell lung cancer (NSCLC) [47], however a perspective use of CRISPR/dCas for epigenetic editing as an anti-cancer strategy requires in depth investigation and testing in combined therapies, as cancer results from a combination of genetic and epigenetic alterations [48].

2. **Immunotherapy enhancement.** CRISPR/Cas9 can be used to engineer T cells to express chimeric antigen receptors [49, 50], to delete inhibitory immune checkpoint expression in T and NK cells [51], and to identify and delete genes involved in drug resistance and enhance treatment efficacy [52–54].

- **Engineering T cells**. CRISPR/Cas9 can be used to edit the genome of T lymphocytes to enhance their ability to recognize and kill cancer cells. This includes engineering T cells to express Chimeric Antigen Receptors (CAR-T), that can specifically target cancer cells. Bispecific T cells are generated by introducing genes that encode TCRs and CARs of the desired specificity and affinity for tumors [49]. CRISPR was successfully used to engineer T cells to express CARs targeting CD19 in patients with B-cell lymphoma leading to durable remissions in a significant proportion of treated patients [55]. CRISPR has been useful in the development of non-autologous CAR-T cells that could be used in any patient without the need for HLA matching, thereby reducing costs and making CAR T-cell therapy accessible to patients lacking sufficient healthy T lymphocytes required for treatment [56].
- **Deleting immune checkpoints**. CRISPR/Cas9 can knock-out genes coding for immune checkpoint proteins, such as *PDCD1* gene, which encodes for PD-1, in primary T lymphocytes [57], or in NK cells [58], and maximize PD-1/Programmed cell Death Ligand 1 (PD-L1) deletion at the genomic level, thereby saving the function of lymphocytes, or NK cells, against tumors. CRISPR/Cas9-mediated disruption of immune checkpoint signaling, such as PD-1, enhances the therapeutic efficacy of CAR-T cells in the immunosuppressive tumor microenvironment [55].

3. **Drug resistance reversal.** The development of therapy resistance is a major obstacle to cancer treatment [59]. Cancer cells resist anticancer drugs by a variety of mechanisms, such as enhancing drugs efflux, enhancing DNA repair, enhancing stemness, inhibiting apoptosis, and altering drug target [60]. Mutations of genes implicated in specific signaling pathways may underlie these events and lead to drug resistance. CRISPR technology allows the identification of genes involved in therapy resistance and their editing to sensitize cancer cells to therapy.

- **Identifying genes involved in resistance to antitumor therapy.** Genome-wide CRISPR knock-out screens technique is used to identify genes involved in drug resistance. CRISPR screens proved that mutations of Polybromo-1 (PBRM1) gene attenuate the effects of EGFR inhibitors in lung cancer by enhancing the continuity of AKT signaling [61]. Gene silencing using the CRISPR/Cas9sgRNA system has revealed that loss of Shugoshin 1 (SGOL1), that plays a crucial role in cell mitosis, may cause resistance to sorafenib in hepatocellular carcinoma [62]. CRISPR/Cas9 screens also defined the importance of the MSH2 gene and mismatch repair in cisplatin resistance in bladder cancer [63] and the role of ABCB1 gene (also named multidrug resistance1, MDR1, which encodes a transmembrane glycoprotein transporting multiple types of chemotherapeutic drugs out of cells) in breast cancer resistance to doxorubicin [64, 65]. The key role of MSH2, Chloride Channel Accessory 2 (CLCA2), and Patched 2 (PTCH2), in the resistance of glioblastoma cells to temozolomide was elucidated using genome-wide CRISPR/Cas9 screen technology [66].
  Poly(ADP-ribose) polymerase (PARP) inhibitors (PARPis) selectively induce synthetic lethality in cancer cells harboring mutations in the BRCA1/2 genes [67]. Genome-wide CRISPR/Cas9 knock-out screens in BRCA1/2-proficient prostate cancer (PC) cells identified genes whose loss has a profound impact on PARPis response and find that loss of Checkpoint Kinase 2 (CHEK2), which is involved in the DNA damage repair response, confers PARPis resistance in PC cells with functional p53 [68], and that deletion of Ribonuclease H2 Subunit B (RNASEH2B), which play a role in DNA replication, confers PC cell sensitivity to PARPis [69].

- **Sensitizing cancer cells to antitumor therapy.** CRISPR/Cas9 can be used to edit genes that contribute to drug resistance, making the cancer cells more susceptible to existing treatments [70]. CRISPR/Cas9 mediated knocking out of glutathione S-Transferase Omega 1 (GSTO1) in colorectal cancer cells enhances the cytotoxicity of chemotherapeutics, including cisplatin and oxaliplatin [71]. CRISPR/Cas9-mediated knock-out of ABCB1/MDR1 gene significantly enhances the chemosensitivity of colorectal cancer cells to doxorubicin (DOX) [65, 72]. Targeting MDR1 using CRISPR/Cas9 system in DOX-resistant breast cancer cells increases drug uptake and intracellular accumulation compared with untreated cells resulting in improved DOX cytotoxicity [64].
  Besides being an immune checkpoint molecule and preventing anti-tumor T cell response, PD-L1 (B7-H1, CD274) has a cancer cell-intrinsic pro-survival function and may contribute to cancer chemoresistance. Activation of multiple signaling pathways promotes multi drug resistance in breast cancer. Notably, activating PhosphatidylInositol 3-Kinase (PI3K)/AKT and mitogen-activated protein kinase (MAPK)/ERK pathways can increase the expression of ABCB1 and enhance DOX resistance [73]. PD-L1 knock-out by CRISPR/Cas9 has proven effective in making triple negative breast cancer cells susceptible to chemotherapy through a reduced activation of p38 MAPK signalling pathway, that is dependent on the association of DNA-PKcs and B7-H1 followed by a downregulation of Bcl-2 [74].

### Applications of CRISPR/Cas9 for molecular diagnostics and prognosis of tumors

CRISPR/Cas9 can be used for early detection and screening of cancer-associated mutations, after capturing circulating tumor (ct) DNA or circulating tumor cells (CTC) in blood samples, obtained through liquid biopsies [75]. This non-invasive approach allows for early cancer detection and monitoring of tumor dynamics over time providing insights into disease progression and potential resistance to therapy. Integrating biosensors, that convert a biological response into an electrical signal, with CRISPR/Cas technology, can enhance the detection of specific cancer-related genetic sequences and identify mutations in oncogenes such as KRAS and EGFR, and tumor suppressor genes, such as TP53 [76]. Genome-wide CRISPR screens can identify new genetic markers associated with tumor aggressiveness, metastasis, and treatment resistance. These markers can be used to predict patient outcomes and personalize anti-cancer therapy. The biosensor can be designed with a sgRNA that targets a specific cancer biomarker sequence. The Cas9 enzyme, guided by the sgRNA, will bind to the target DNA sequence if present. The binding event can trigger a fluorescence change, an electrochemical signal, or a colorimetric change. These biosensors allow early rapid and sensitive detection of cancer by identifying the specific associated mutations or gene sequences [77]. Cas effectors in the Cas9, Cas12, Cas13 and Cas14 families have been used for biosensing purpose [78–80].

To provide more precise or conclusive information for molecular diagnostics, multiplex detection of several targets from a single tumor sample is currently in development [81, 82].

### Applications of CRISPR/Cas9 to investigate the role of genes involved in carcinogenesis by functional genomics

CRISPR/Cas9 system can be used to understand gene function, at the single-cell level or in the context of an entire organism, observing the effects of its loss (gene knock-out), or the consequences of insertion or replacement of a specific gene or genetic sequence (gene knock-in) or the significance of modulating gene expression without altering the DNA sequence (gene activation, CRISPRa, and gene interference, CRISPRi) using dCas9 (which binds to, but does not cut DNA) fused with activation (VP64, p300) or repression (KRAB) domains to upregulate or downregulate target genes [83]. Selective activation or repression of genes can be used to investigate their impact on tumor growth and patient outcomes. Genome-wide screen by CRISPRa library in vivo for drivers of hepatocellular carcinoma (HCC) progression revealed that overexpression of XAGE1B, PLK4, LMO1 and MYADML2 genes promoted HCC cell proliferation and invasion, which were suppressed by their inhibition. Furthermore, high level of MYADML2 protein was associated with worse overall survival and reduced sensitivity to chemotherapeutic drugs [84].

CRISPR/Cas9 enables genome library screening approach, which can detect genes essential for cancer cell survival and drug resistance *via* Gain-Of-Function (GOF) or Loss-Of-Function (LOF). Genome-wide CRISPR/Cas9-mediated LOF screens in tumor and metastasis have discovered key genes involved in early, late and metastatic cancer [85, 86]. CRISPR/Cas9-based high-throughput LOF screening has identified farnesyltransferase among the top hits contributing to sunitinib resistance in clear cell renal cell carcinoma [87]. CRISPR/Cas9 screening platform have been used to identify genetic vulnerabilities in acute myeloid leukemia (AML) cells, including several established (such as DOT1L, BCL2, and MEN1) and novel (such as KAT2A) therapeutic targets for AML [88]. In vivo, genome-wide CRISPR LOF screen has identified TGFβ3 as an actionable biomarker of palbociclib resistance in triple negative breast cancer [89]. Genome-wide CRISPR screen in glioblastoma has identified loss of IFNγR signalling as a mechanism of resistance to CAR-T cell cytotoxicity [90]. Genome-scale CRISPR/Cas9 screening of 324 cancer cell lines from 30 cancer types, developed to provide an effective portfolio of cancer drug targets, has recently identified Werner syndrome ATP-dependent helicase (WRN) as a new candidate synthetic lethal target in microsatellite instability tumors [91].

In conclusion, CRISPR/Cas9 system can be used to identify cancer driving mutations, prognostic biomarkers [92–94] and therapeutic target genes, which are essential for precision medicine and personalized management of cancer patients.

### Challenges to address

While the potential of CRISPR/Cas9 in cancer treatment and diagnosis is immense, several challenges remain. Issues to be addressed and strategies to overcome limitations of the CRISPR/Cas9 system in clinical applications involve: **(a)** **Delivery methods**. Efficient and targeted delivery of the CRISPR components (Cas9 protein and sgRNA) to specific cells and tissues is crucial. Current methods, such as viral vectors (e.g., AAV, lentivirus) and non-viral approaches (e.g., lipid nanoparticles), have limitations in terms of specificity, efficiency, and safety. *Natural occurring AAV variants*, which can be isolated from human and nonhuman primate tissues using high-cycle PCR and high-throughput sequencing [95] and *directed evolution* (which is an engineering approach to develop novel AAV variants with designated specificity, including random or defined peptide insertion, capsid shuffling, error-prone PCR, and saturation mutagenesis) are strategies currently employed for viral vector optimization which may reduce genotoxicity, and improve safety, tissue specificity, and packaging capacity [96]. Non-viral vectors, such as, *lipid nanoparticles*, which can encapsulate CRISPR/Cas9 components (and has proven particularly useful for delivering mRNA or protein forms of CRISPR/Cas9) and facilitate cellular uptake through endocytosis [97], and *polymeric nanoparticles*, which can be engineered to release their payload in response to specific stimuli (e.g., pH, enzymes) [98], can be functionalized with antibodies that specifically recognize and bind to tumor-associated antigens [99]. *Gold nanoparticles* offer a high degree of surface modification, allowing for the attachment of various targeting ligands [100]. *Naturally occurring exosomes and extracellular vesicles*, can carry CRISPR/Cas9 components to specific tissues, minimizing immune responses and reducing off-target effects [101–103]. Exosome-based CRISPR delivery systems are being studied for their ability to overcome blood-brain barrier and blood-brain tumor barrier to target glioblastoma cells [104, 105]; **(b) Off-target effects**. CRISPR can sometimes cut DNA at unintended sites, leading to off-target effects that cause unintended genetic modifications, which can be harmful and potentially increase cancer risks or create unwanted immune responses. Improving the specificity of the CRISPR system is essential to minimize these risks and can be achieved with the following methods: *i) Enhancing HDR efficiency* with small molecules, such as HDR enhancers (e.g., RS-1, a stimulator of RAD51, a DNA repair protein, ref. [106]), to increase the proportion of cells undergoing HDR [107]; *ii) Using cell cycle regulators* (cyclin-dependent kinase inhibitors, checkpoint kinase 1 inhibitors, aurora kinase inhibitors, such as VX-680 – Tozasertib, which block cells in the G2/M phase) to transiently push cells into HDR-active phases and make CRISPR/Cas9 editing less effective in non-dividing or slowly dividing cells [108]; *iii) Improving sgRNA design* by incorporating modified nucleotides or chemical modifications in sgRNAs [109] to enhance binding specificity and stability [110], as obtained in human primary T cells and CD34^+^ hematopoietic stem and progenitor cells [111], and by using artificial intelligence (AI)-powered bioinformatics tools, such as DeepCRISPR [112] and CRISPR-DO [113, 114]. These machine learning algorithms have been developed to predict the efficiency and specificity of guide RNAs and are instrumental in designing gRNAs that maximize on-target efficacy and minimize off-target risks, leading to safer CRISPR applications, especially for therapeutic uses; *iv) Improving the enzyme’s specificity* by using high-fidelity Cas9 variants, like eSpCas9 [115], HypaCas9 [116] and SpCas9-HF1 [117], which have been engineered (*via* mutations that reduce their binding to off-target sites) to reduce unintentional cuts in the genome and prevent unintended genetic changes [118, 119]; *v) Using base editors*, like CRISPR/Cas9 fused to deaminases, which enable direct chemical alteration of a single DNA base without creating double-stranded breaks, allowing for precise editing at the single-base level which is crucial for treating point mutations [120]; *vi) Applying prime editing*, which has been developed to generate targeted point mutations, as well as insertions or deletions in an HDR-independent manner [121] and consists of a prime editing guide RNA and a fusion protein construct composed of Cas9 nickase (a modified version of the CRISPR/Cas9 protein, engineered to create single-strand “nicks” in DNA rather than the typical double-strand break, DSB, that standard Cas9 produces) with an inactivated HNH domain and an engineered reverse transcriptase domain [122]. This approach reduces the likelihood of off-target effects and offers a broader range of genetic modifications [123]; *vii) Applying advanced screening techniques for off-target detection*, such as GUIDE-seq [124] and Digenome-seq [125], which are sequencing-based methods used to identify off-target effects by mapping unintended DNA cuts. GUIDE-seq and Digenome-seq help evaluate the precision of CRISPR edits and refine gRNA designs to reduce off-target activity. CHANGE-seq is a simplified, sensitive and automatable tagmentation-based method for measuring the genome-wide activity of Cas9 to understand the specificity of genome editors [126]. CIRCLE-seq is a newer, rapid and more sensitive technique that detects off-target effects by sequencing circular DNA fragments, providing an accurate measure of unintended DNA breaks [127, 128]; **(c) Immune Response to CRISPR Components.** Cas9 derived from bacterial species like *Streptococcus pyogenes* (SpCas9) and *Staphylococcus aureus* (SaCas9), which the human immune system may recognize as foreign (through pattern recognition receptors (PRRs), triggering inflammation and cytokine release) leads to unwanted innate and adaptive cellular immune responses [129]. The risk of such adverse reactions can be minimized by *1. Using less immunogenic Cas9 variants* from less common bacterial strains [130], which can be selected with prediction models [131], or orthologs with low immunogenicity from non-pathogenic bacteria, such as Cas9 from *Geobacillus stearothermophilus*, which was found to be highly stable in human plasma [132]. *2.* *Delivering Cas9 transiently*, e.g., as mRNA or protein, rather than DNA, limits its expression duration, reducing exposure to the immune system [133, 134]. *3. Humanizing Cas9 Protein*, through the incorporation of sequences or motifs that mimic human proteins and can reduce immune recognition [135, 136]. *4. Using less immunogenic delivery systems*, such as lipid nanoparticles or polymer-based carriers to shield Cas9 from immune recognition [97–99]; **(d) Ethical and regulatory issues**. Although developing anti-cancer treatments using CRISPR focus on somatic cells, ensuring that CRISPR is restricted to these cells can be challenging. Germline editing, which would be passed on to future generations, raises ethical concerns. Strong ethical guidelines and regulatory frameworks, possibly overseen by international bodies like the WHO, need to be established to ensure the safe and ethical use of CRISPR in humans [137, 138]. Transparent public engagement, that involves encouraging open dialogue with the public about the benefits, risks, and ethical issues of CRISPR technology, can improve understanding and trust of potential patients. Prioritizing research on CRISPR technologies and promoting health policies for equitable access to cutting-edge treatments and diagnostics could reduce social disparities in accessing up-to-date health care.

Current clinical trials testing CRISPR/Cas9-based antitumor therapeutic strategies are reported in Table 2 [139]. Meanwhile, research applying CRISPR systems based on different enzymatic proteins, such as Cas12 and Cas 13, is gaining ground.

**Table 2:** Active clinical trials testing therapeutic approaches based on the CRISPR/Cas9 system for cancer treatment [139]

NCT Number	Study Title	Study Status	Conditions	Sponsor	Phases
NCT04035434	A Safety and Efficacy Study Evaluating CTX110 in Subjects With Relapsed or Refractory B-Cell Malignancies (CARBON)	RECRUITING	B-cell Malignancy|Non-Hodgkin Lymphoma|B-cell Lymphoma|Adult B Cell ALL	CRISPR Therapeutics AG	Phase 1/2
NCT04037566	CRISPR (HPK1) Edited CD19-specific CAR-T Cells (XYF19 CAR-T Cells) for CD19 + Leukemia or Lymphoma.	RECRUITING	Leukemia Lymphocytic Acute (ALL) in Relapse|Leukemia Lymphocytic Acute (All) Refractory|Lymphoma, B-Cell|CD19 Positive	Xijing Hospital	Phase 1
NCT04244656	A Safety and Efficacy Study Evaluating CTX120 in Subjects With Relapsed or Refractory Multiple Myeloma	ACTIVE_NOT_RECRUITING	Multiple Myeloma	CRISPR Therapeutics AG	Phase 1
NCT04417764	TACE Combined With PD-1 Knockout Engineered T Cell in Advanced Hepatocellular Carcinoma.	RECRUITING	Advanced Hepatocellular Carcinoma	Central South University	Phase 1
NCT04426669	A Study of Metastatic Gastrointestinal Cancers Treated With Tumor Infiltrating Lymphocytes in Which the Gene Encoding the Intracellular Immune Checkpoint CISH Is Inhibited Using CRISPR Genetic Engineering	RECRUITING	Gastrointestinal Epithelial Cancer|Gastrointestinal Neoplasms|Cancer of Gastrointestinal Tract|Cancer, Gastrointestinal|Gastrointestinal Cancer|Colo-rectal Cancer|Pancreatic Cancer|Gall Bladder Cancer|Colon Cancer|Esophageal Cancer|Stomach Cancer	Intima Bioscience, Inc.	Phase 1/2
NCT04438083	A Safety and Efficacy Study Evaluating CTX130 in Subjects With Relapsed or Refractory Renal Cell Carcinoma (COBALT-RCC)	ACTIVE_NOT_RECRUITING	Renal Cell Carcinoma	CRISPR Therapeutics AG	Phase 1
NCT04502446	A Safety and Efficacy Study Evaluating CTX130 in Subjects With Relapsed or Refractory T or B Cell Malignancies (COBALT-LYM)	RECRUITING	T Cell Lymphoma	CRISPR Therapeutics AG	Phase 1
NCT04557436	TT52CAR19 Therapy for B-cell Acute Lymphoblastic Leukaemia (B-ALL)	ACTIVE_NOT_RECRUITING	B Acute Lymphoblastic Leukemia	Great Ormond Street Hospital for Children NHS Foundation Trust	Phase 1
NCT04637763	CRISPR-Edited Allogeneic Anti-CD19 CAR-T Cell Therapy for Relapsed/Refractory B Cell Non-Hodgkin Lymphoma (ANTLER)	RECRUITING	Lymphoma, Non-Hodgkin|Relapsed Non Hodgkin Lymphoma|Refractory B-Cell Non-Hodgkin Lymphoma|Non Hodgkin Lymphoma|Lymphoma|B Cell Lymphoma|B Cell Non-Hodgkin’s Lymphoma	Caribou Biosciences, Inc.	Phase 1
NCT04976218	TGFÎ²R-KO CAR-EGFR T Cells in Previously Treated Advanced EGFR-positive Solid Tumors	RECRUITING	Solid Tumor, Adult|EGFR Overexpression	Chinese PLA General Hospital	Phase 1
NCT05309733	A Long-term Follow-up Study of Patients Who Received VOR33	RECRUITING	Leukemia, Myeloid, Acute	Vor Biopharma	Phase 2
NCT05397184	Base Edited CAR7 T Cells to Treat T Cell Malignancies (TvT CAR7)	RECRUITING	Relapsed/Refractory T-cell Acute Lymphoid Leukaemia	Great Ormond Street Hospital for Children NHS Foundation Trust	Phase 1
NCT05447169	Epstein-Barr Virus Antibody and Epstein-Barr Virus DNA for Nasopharyngeal Carcinoma Screening	RECRUITING	Nasopharyngeal Carcinoma	Sun Yat-sen University	
NCT05566223	CISH Inactivated TILs in the Treatment of NSCLC	NOT_YET_RECRUITING	Carcinoma, Non-Small-Cell Lung|Metastatic Non Small Cell Lung Cancer|Stage IV Non-small Cell Lung Cancer|Squamous Cell Lung Cancer|Adenocarcinoma of Lung|Large Cell Lung Cancer	Intima Bioscience, Inc.	Phase 1/2
NCT05631912	TRAC Locus-inserted CD19-targeting STAR-T Cell Therapy in r/r B-NHL	RECRUITING	Non-hodgkin Lymphoma, B Cell	Chinese PLA General Hospital	Phase 1/2
NCT05643742	A Safety and Efficacy Study Evaluating CTX112 in Subjects With Relapsed or Refractory B-Cell Malignancies	RECRUITING	B-cell Lymphoma|Non-Hodgkin Lymphoma|B-cell Malignancy|Chronic Lymphocytic Leukemia (CLL)/Small Lymphocytic Lymphoma (SLL)|Follicular Lymphoma|Mantle Cell Lymphoma|Marginal Zone Lymphoma|Large B-cell Lymphoma	CRISPR Therapeutics AG	Phase 1/2
NCT05662904	Genetic Ablation of CD33 in HSC to Broaden the Therapeutic Index of CD33-directed Immunotherapy in Patients With AML	NOT_YET_RECRUITING	Relapsed/Refractory Acute Myeloid Leukemia (AML)	German Cancer Research Center	Phase 1
NCT05722418	CRISPR-Edited Allogeneic Anti-BCMA CAR-T Cell Therapy in Patients With Relapsed/Refractory Multiple Myeloma	RECRUITING	Relapsed/Refractory Multiple Myeloma	Caribou Biosciences, Inc.	Phase 1
NCT05795595	A Safety and Efficacy Study Evaluating CTX131 in Adult Subjects With Relapsed or Refractory Solid Tumors	RECRUITING	Clear Cell Renal Cell Carcinoma|Cervical Carcinoma|Esophageal Carcinoma|Pancreatic Adenocarcinoma|Malignant Pleural Mesothelioma	CRISPR Therapeutics AG	Phase 1/2
NCT06014073	TRAC and Power3 Genes Knock-out Allogeneic CD19-targeting CAR-T Cell Therapy in r/r B-NHL	RECRUITING	Non Hodgkin’s Lymphoma	Chinese PLA General Hospital	Phase 1/2
NCT06128044	CRISPR-Edited Allogeneic Anti-CLL-1 CAR-T Cell Therapy in Patients With Relapsed/Refractory Acute Myeloid Leukemia	RECRUITING	Acute Myeloid Leukemia, in Relapse|Acute Myeloid Leukemia Refractory	Caribou Biosciences, Inc.	Phase 1

## Opportunities for cancer diagnosis and treatment using the novel Cas12- and Cas13-based CRISPR systems

Discovery of alternative CRISPR systems beyond the widely used Type II *Streptococcus pyogenes* Cas9 has expanded the toolkit for genetic manipulation.

CRISPR/Cas12 is a member of the CRISPR gene-editing family, which belongs to the Class 2, Type V system, with Cas12a being one of its most used subtypes [3]. Cas12 recognizes complementary ssDNA or dsDNA containing a PAM sequence under the guidance of its CRISPR RNA (crRNA), which directs DNA cleavage through a single RuvC structural domain [140]. Cas12 has been reported to have efficient trans-cleavage activity. It can induce robust and nonspecific ssDNA cleavage when it cleaves dsDNA in a sequence-specific manner, a property that has been widely used, especially in diagnostic applications, since its discovery in 2018 [141]. CRISPR/Cas12 can induce double-stranded breaks (DSBs) for permanent gene knock-out, insertion, or correction (Fig. 1b). Compared to Cas9, Cas12 has important advantages, such as: *1. generating staggered or “sticky” ends in DNA*, which can enhance homology-directed repair and be advantageous in precision genome editing [142]; *2. targeting several distinct genomic loci*, a process referred to as multiplexing, which enables strategies for targeting diseases involving multiple genes [143]; *3. the property of non-specific single-strand DNA cleavage activity*, which makes Cas12 suitable for molecular diagnostics, such as detecting specific DNA sequences in infectious diseases and cancers [144].

CRISPR/Cas13 is another member of the CRISPR family of technologies, but it stands out because it targets RNA rather than DNA. Cas13, which belongs to the Class 2, Type VI system, contains the HEPN structural domain that binds to RNA for cleavage and is guided by a single RNA [145]. Cas13a (formerly C2c2) is one of its widely used subtypes [146]. It has been found that the Cas13a-crRNA complex, once activated by binding to its target RNA, cleaves it as well as other non-specific RNAs. This trans-cleavage capability expands the range of targets to be detected [19]. Since Cas13, unlike Cas9, binds to and cleaves single-stranded RNA molecules in a sequence-specific manner, it can be safely used in situations where temporary gene expression regulation is desired, without altering the host genome [103, 147] (Fig. 1c), which makes Cas13 a versatile tool for detecting viral- or cancer-related RNA sequences for molecular diagnostics [147]. Compared to Cas9, which typically affects only its target DNA site directly and does not have collateral activity, Cas13 has a unique collateral activity meaning that after it binds to its target RNA and being activated, it can nonspecifically cleave other RNAs nearby, a feature exploited in sensitive diagnostic techniques, but to be carefully managed in therapeutic applications [refs 4, 148].

Combined applications of Cas12 and Cas13, in tandem, are feasible and allow researchers to edit DNA and modulate RNA within the same system. CRISPR/Cas12 and CRISPR/Cas13 expand the toolkit available for genetic and molecular biology research, offering new possibilities for genome and transcriptome editing, as well as innovative diagnostic applications.

## CRISPR/Cas12

CRISPR/Cas12 is an RNA-guided endonuclease similar to Cas9, with some key differences. CRISPR/Cas12a (Cpf1) is a CRISPR effector that controls target genes by recognizing thymine-rich PAM sequences. Cas12a has a higher sensitivity to mismatches in the guide RNA than does Cas9, therefore, off-target sequence recognition and cleavage are lower [148]. ***The main features of CRISPR/Cas12 system are the following***: ***(a) Single-strand nicking***. Cas12 can create a single-strand break or “nick” in the DNA, which can be useful for more precise genome editing and reduced off-target effects compared to double-stranded breaks; ***(b) PAM sequence***. Cas12 recognizes a different PAM sequence than Cas9, expanding the range of targetable DNA sequences; ***(c) Collateral cleavage activity.*** Once activated by binding to the target DNA, Cas12 exhibits nonspecific cleavage activity on ssDNA. This property is harnessed in diagnostic applications [149].

***Applications of CRISPR/Cas12 system include***:*** (a) Genome editing***. Similarly to Cas9, Cas12 can be used for gene knock-outs, insertions, or substitutions. Its ability to make single-stranded nicks allows for more precise edits; ***(b) Gene regulation.*** Cas12 can be modified to regulate gene expression without cutting DNA, by using dCas12, a catalytically inactive nuclease; ***(c) Diagnostics.*** The collateral cleavage activity of Cas12 is used in diagnostic assays like DETECTR (DNA Endonuclease Targeted CRISPR Trans Reporter), where it can detect specific DNA sequences, with high sensitivity, by cleaving reporter molecules upon target recognition [150].

## Applications of CRISPR/Cas12 in cancer research and treatment

Unlike Cas9, Cas12 can cleave both ssDNA and dsDNA, and it has unique properties that can be advantageous for certain applications in oncology, such as those described below.

1. **Gene editing.** CRISPR/Cas12 can be used to edit genes that are involved in cancer development and progression. The intra-tumoral delivery of an oncolytic Adenovirus (oAd) co-expressing Cas12a and a crRNA targeting the *Epidermal Growth Factor Receptor (EGFR)* gene (oAd/Cas12a/crEGFR) in lung cancer cells has proven to induce efficient and precise editing of the targeted *EGFR* gene in a cancer-specific manner, without detectable off-target nuclease activity and elicited a potent antitumor effect *via* induction of apoptosis and inhibition of proliferation in cancer cells, ultimately leading to tumor regression [151]. The multiplex gene targeting capability of Cas12a were exploited to delete three frequently mutated genes, such as TP53, Adenomatous polyposis coli (APC), and Phosphatidylinositol-4,5-bisphosphate 3-kinase catalytic subunit alpha gene (PIK3CA) simultaneously, in colorectal cancer patients [152]. CRISPR/Cas multiplex biosensing is currently being developed by coupling different Cas12a-crRNA complexes, which detect several targets in parallel from a single tumor sample, with conversion of targeted information into colorimetric signals [153].These studies have highlighted the unique advantages of Cas12a in therapeutic editing for cancer cells.
2. **Targeted therapies.** CRISPR/Cas12 can be used to develop targeted cancer therapies. For instance, it can be used to introduce the CAR gene into T cells at specific locations within the genome and generate CAR-T cells with enhanced specificity and efficacy against cancer cells [154]. Beyond introducing the CAR gene, CRISPR/Cas12 can be used to knock-out the PD-1 gene preventing T cells from becoming exhausted and allowing them to maintain their cancer-fighting activity for longer periods [155]. Traditional methods of producing CAR-T cells often involve viral vectors, which can integrate randomly into the genome, potentially leading to mutations and cancer. CRISPR/Cas12 provides a more controlled and safer alternative, with the ability to target specific genes with minimal off-target effects [156].
3. **Synthetic lethality (SL) approaches.** Synthetic lethality occurs when the simultaneous impairment of two genes leads to cell death, whereas the dysfunction of either gene alone does not. In cancer therapy, this concept is used to selectively kill cancer cells by targeting a gene that becomes essential for survival due to the mutation or loss of another gene that is already compromised in the cancer cell. In tumor harboring a mutation in one of these genes, targeting the partner gene, which is functional, can induce death in cancer cells, but leave unaffected normal cells, in which neither gene is typically mutated. **CRISPR/Cas12 can be used to identify synthetic lethality interactions** (SLI) in cancer cells, by targeting specific genes that, when simultaneously inhibited along with existing mutations, can lead to cancer cell death while sparing normal cells [157]. By identifying synthetic lethal partners of mutations that are common in certain cancers **CRISPR/Cas12 can help identify new drug targets.** For instance, BRCA1 or BRCA2 mutations in breast and ovarian cancers. Since PARP enzymes are involved in repairing single-strand breaks in DNA, inhibiting PARP in cells with BRCA1 or BRCA2 mutations leads to the accumulation of DNA damage, ultimately causing cell death. Ataxia Telangiectasia and Rad3-related protein, ATR, and Checkpoint Kinase 1, CHK1, are involved in DNA damage response. In TP53-mutant ovarian cancer cells, targeting the ATR/CHK1 pathway can lead to an accumulation of DNA damage and cell death, making ATR and CHK1 inhibitors promising therapeutic targets [158]. Drugs that inhibit the synthetic lethal partner gene could selectively kill cancer cells while sparing normal cells. **CRISPR/Cas12 can be used to identify effective combination therapies** by revealing which gene knock-outs (and thus potential drug targets) are synthetic lethal with existing cancer therapies [159]. This can lead to the development of combination treatments that are more effective and have fewer side effects.
4. **Cancer diagnostics.** Cas12 has been harnessed for diagnostic purposes, due to its collateral cleavage activity, where it non-specifically cleaves ssDNA upon activation. This property is exploited in diagnostic assays to detect cancer biomarkers with high sensitivity and specificity [160, 161]. Cas12 can be programmed to detect specific mutations in oncogenes or tumor suppressor genes associated with different types of cancer. For instance, Cas12-based assays can identify mutations in genes like KRAS [162], TP53 [160], and BRAF [163], which are commonly mutated in cancers such as lung, colon, and melanoma. Cas12 can detect even low concentrations of ctDNA in liquid biopsies with high specificity, enabling early detection of cancer and monitoring of minimal residual disease. Coupled with an electrochemical biosensor with a low detection limit of 2 cells/mL, CRISPR/Cas12a system has been used to develop highly sensitive nucleic acid-based analytical [164].

In summary, CRISPR/Cas12 represents a powerful tool in advancing knowledge on the molecular basis of cancer, with potential applications ranging from gene editing and diagnostics to the development of targeted therapies. However, its clinical implementation requires overcoming technical, ethical, and regulatory hurdles.

## DETECTR for cancer detection

DNA Endonuclease-Targeted CRISPR Trans Reporter (DETECTR) is another CRISPR-based diagnostic platform, akin to Specific High Sensitivity Enzymatic Reporter UnLOCKing (SHERLOCK), developed for the rapid and accurate detection of specific nucleic acid sequences [150]. Leveraging the CRISPR/Cas12 system, DETECTR, which achieves attomolar sensitivity for DNA detection, has shown great potential in different applications, such as infectious disease diagnostics, genetic testing and cancer diagnostics [79].

***The main features of DETECTR assay are the following: (a)*** *Signal amplification.* The collateral cleavage activity of Cas12 enzyme, that indiscriminately cuts nearby ssDNA reporters, results in amplified signal detection, enhancing sensitivity and enabling the detection of low-abundance targets, tumor DNA, or cancer-related viruses, in tissue samples and bodily fluids (e.g., blood, saliva, urine) from patients, facilitating early diagnosis and enabling precision medicine approaches. DETECTR can be adapted to detect various cancer-related mutations, such as those in oncogenes (e.g., KRAS, BRAF) or tumor suppressor genes (e.g., TP53), after detecting circulating tumor DNA (ctDNA) in blood samples. For viruses associated with cancer (e.g., Human Papillomavirus (HPV16 and HPV18) [165], Hepatitis B Virus (HBV), or Epstein-Barr Virus (EBV), the DETECTR assay targets specific viral sequences. Similarly to tumor DNA detection, the Cas12a protein is guided to the viral DNA or RNA sequence. Upon recognition, Cas12a is activated [166]. DETECTR can be tailored to detect multiple biomarkers simultaneously, providing a personalized profile of a patient’s cancer; ***(b)*** *Specificity for target sequence and programmable specificity.* The use of CRISPR/Cas12a ensures that the assay specifically detects the intended target without cross-reactivity with non-target sequences, reducing false positives [165]. The guide RNA (gRNA) used in the DETECTR is customizable and allows the assay to be programmed to recognize virtually any nucleic acid sequence and detect specific targets of interest across different applications; ***(c)*** *Speed and simplicity.* DETECTR provides accurate and rapid results, often within an hour or even minutes, which allows for faster decision-making in clinical settings. Furthermore, it can be performed with relatively simple equipment, low estimated cost and sustainable cost-benefit ratio. This makes it suitable for both clinical laboratories and point-of-care settings [165]; ***(d) ****Versatility.* Like SHERLOCK, DETECTR can be adapted to detect a variety of nucleic acid targets, making it applicable for multiple types of cancer biomarkers. While DETECTR is primarily designed for DNA detection, it can also be adapted for RNA targets by incorporating a reverse transcription step, converting RNA to complementary DNA (cDNA) before detection [167]; ***(e)*** *Monitoring disease progression and treatment response*. By tracking the levels of specific cancer-related DNA mutations or methylation patterns during treatment, DETECTR can help assess the effectiveness of therapy and detect early signs of recurrence.

Despite the advantages it could provide, several challenges must be addressed to move the DETECTR platform into the clinical setting, such as: 1. carrying out clinical trials and validation studies to confirm the accuracy, the sensitivity, and the specificity of DETECTR for different types of cancer and patient populations; 2. integration of DETECTR within the existing diagnostic frameworks and compliance with regulatory standards; 3. ensuring the reliability of DETECTR addressing potential issues with false positives and negatives, which can impact clinical decisions.

In conclusion, DETECTR represents a significant advancement in cancer diagnostics, offering a rapid, sensitive, and cost-effective method for detecting cancer-related genetic mutations and biomarkers. Its application in non-invasive liquid biopsies and real-time monitoring of disease progression holds the promise of improving early cancer detection, treatment outcomes, and overall patient care.

### CRISPR/Cas13

CRISPR/Cas13 is an RNA-guided RNA endonuclease that specifically targets RNA molecules instead of DNA. Upon binding to a target RNA sequence, Cas13 then cleaves the RNA, which can be used for various applications involving RNA manipulation [148].

***The main features of CRISPR/Cas13 system are the following: (a) RNA targeting***. Unlike Cas9 and Cas12, which target DNA, Cas13 specifically binds to and cleaves RNA molecules. This makes it an excellent tool for post-transcriptional regulation; ***(b) Collateral cleavage activity***. Similarly to Cas12, Cas13 also exhibits collateral cleavage activity, but it targets RNA instead of DNA, which makes it useful for RNA detection assays [148]; ***(c) Programmability***. Cas13 can be programmed with a specific guide RNA to target any RNA sequence, allowing the precise manipulation of RNA.

***Applications of CRISPR/Cas13 system include: (a) RNA knock-down***. Cas13 can be used to degrade specific RNA molecules, thereby reducing the expression of target genes at the RNA level. This property is useful for studying gene function and for therapeutic applications where reduction of harmful RNA is desired [168]; ***(b) RNA editing.*** By fusing Cas13 with RNA-editing enzymes, researchers can achieve precise RNA editing, which can be beneficial for correcting RNA transcripts without altering the genome [168]; ***(c) Diagnostics.*** The collateral cleavage activity of Cas13 can be exploited in diagnostic platforms like SHERLOCK, which can detect specific RNA sequences with high sensitivity and specificity. This feature allows the detection of viral RNA, as demonstrated for the detection of severe acute respiratory syndrome coronavirus 2 (SARS‑CoV‑2) [160].

## Applications of CRISPR/Cas13 in cancer research and treatment

CRISPR/Cas13 has garnered significant attention for its potential applications in cancer research and treatment [169]. Unlike the more widely known CRISPR/Cas9, which targets DNA, CRISPR/Cas13 targets RNA, enabling the following approaches for cancer therapeutics and diagnostics.

1. ***Knock-down of oncogenes and drug-resistant genes for therapeutic and discovery purposes.***

- *Therapeutic targeting of oncogenes and non-coding RNAs.* By degrading the mRNA transcripts of oncogenes, CRISPR/Cas13 can reduce the expression of proteins that drive cancer cell proliferation and survival. Cas13a-based CRISPR system has proven effective in knocking down the expression of oncogenes, such as *TERT*, *EZH2*, and *RelA*, at both the mRNA and protein levels, in human hepatocarcinoma cells and tumor xenografts, leading to significant cancer cell apoptosis and tumor growth inhibition [170].
  CRISPR/Cas13a-mediated knock-down of oncogenic KRAS^G12D^ mRNA expression in pancreatic cancer cells, revealed up to a 94% knock-down efficiency, with no detectable effects on wild-type KRAS mRNA, resulting in cancer cell apoptosis and marked tumor shrinkage [171]. These data support the use of CRISPR/Cas13a system as a flexible, targeted therapeutic tool.
  Cas13 can also target non-coding RNAs, such as long non-coding RNAs (lncRNAs) and microRNAs (miRNAs), which play crucial roles in cancer progression [172, 173].

b. *Identifying cancer driver genes and therapeutic targets.* CRISPR/Cas13 can be used in high-throughput screens to knock-down genes across the genome, helping identify genes that are essential for cancer cell survival [174]. CRISPR/Cas13-based knock-down screens in breast cancer cells has been used to identify breast cancer-risk associated lncRNAs, such as KILR, which, when knocked down, promotes DNA replication and cancer cell proliferation, when overexpressed, leads to cancer cell apoptosis [175].
c. *Identifying drug-resistant genes and mutations at the RNA level.* Especially used to identify RNA transcripts from drug-resistant bacteria, viruses, or parasites by targeting specific resistance genes (e.g. those encoding beta-lactamases, efflux pumps, or viral polymerase mutations), Cas13 can also detect mRNA transcripts that relate to drug resistance in cancer cells and provides evidence supporting their biological significance. For instance, Cas13 could be used to detect deregulation of cAMP-regulatory element-binding protein (CREB) associated with resistance to carboplatin in metastatic breast cancer [176], multidrug resistance-associated protein 3 (MRP3) and multidrug resistance 1 (MDR1) genes associated to carboplatin resistance in NSCLC [176], and NRF2, TGFβ and TPp53 signaling associated with platinum-based chemotherapy resistance in ovarian cancer [176].

2. ***Detection of specific RNA sequences associated with cancer for diagnostic applications.*** CRISPR/Cas13 system can be engineered to detect specific RNA sequences associated with cancer in the context of circulating (ct)RNA obtained from liquid biopsies (e.g. blood, urine) providing a minimally invasive method for early cancer detection and monitoring. The SHERLOCK platform, which utilizes CRISPR/Cas13, can detect cancer-related RNAs with high sensitivity and specificity [177].
  CRISPR/Cas13’s ability to target RNA opens new avenues for gene downregulation, functional genomics, and diagnostics with potential benefit for public health [178, 179]. Overcoming the challenges related to delivery, specificity, and clinical translation is essential for realizing its full potential in oncology.

## SHERLOCK for cancer detection

Originally developed for detecting viral and bacterial infections, SHERLOCK is an innovative diagnostic platform which uses CRISPR/Cas13 to detect cancer-related RNAs with high sensitivity and specificity [93, 177].

**The main features of SHERLOCK assay are the following**: ***(a) ****High sensitivity and specificity*. SHERLOCK uses CRISPR/Cas systems, particularly Cas13 and Cas12, which can be programmed to target specific RNA or DNA sequences. It can detect and amplify very small quantities of nucleic acids (such as DNA or RNA) and identify genetic mutations associated with cancer enabling early diagnosis and accurate monitoring of treatment response. SHERLOCK’s sensitivity allows for the detection of cancer-related biomarkers in non-invasive liquid biopsies, such as blood, urine, or saliva [177]; ***(b)*** *Versatility*. SHERLOCK’s flexibility lies in its ability to detect a wide range of nucleic acids, including DNA, RNA, and microRNAs (miRNAs), which are key biomarkers in cancer. Different cancers are associated with specific genetic mutations, gene expression profiles, and epigenetic changes. SHERLOCK can be programmed to target and identify these diverse biomarkers, enabling the detection of multiple cancer types, even at early stages. One of SHERLOCK’s strengths is its ability to simultaneously detect multiple targets in a single assay, a process known as multiplexing. This feature is particularly useful in cancer diagnosis, where a single cancer type may be associated with several genetic mutations or alterations. By detecting multiple biomarkers in parallel, SHERLOCK can provide a more comprehensive profile of the cancer, aiding in more accurate diagnosis and personalized treatment planning; ***(c) ****Rapid results and cost-effectiveness*. SHERLOCK can deliver results quickly, making it suitable for both clinical and point-of-care settings. Rapid detection is critical for early cancer diagnosis and timely treatment. Compared to traditional diagnostic methods like sequencing or imaging, SHERLOCK does not require extensive infrastructure or expensive equipment, and offers a more affordable option, improving accessibility to cancer screening and diagnostics.

### Perspectives and challenges to overcome to move the SHERLOCK platform into the clinical setting

As required for the routine use of DETECTR, clinical trials and validation studies are needed to standardize SHERLOCK for cancer diagnostics and to ensure its reliability across diverse patient populations. As for DETECTR, integration within existing diagnostic frameworks along with scalability and accessibility are issues that need to be addressed in the near future. A summary of common features and differences between the two platforms are provided in Table 3.

**Table 3:** Applications and characteristics of DETECTR and SHERLOCK diagnostic systems

Method	Enzyme	Target	Mechanism of action	Specificity	Sensitivity	Duration of a single assay	Applications
***DETECTR***	Cas12a (Cpf1)	DNA	DETECTR uses Cas12a, which, upon binding to its target dsDNA, can cleave not only its specific target, but also any nearby ssDNA reporter molecule. The ssDNA reporters are labelled with a fluorescent or colorimetric dye which, when cleaved, produce a detectable signal.	6 nucleotide	1 × 10^− 18^ M	2 h	Detection of viral DNA in clinical samples (*human papillomavirus*) and cell cultures.
***SHERLOCK***	Cas13a (C2c2) Cas12a (Cpf1)	RNA DNA	SHERLOCK utilizes Cas13a, which, upon binding to its target RNA, can also cleave nearby ssRNA reporters. Similar to DETECTR, the ssRNA reporters are labelled with a fluorescent or colorimetric dye, producing a detectable signal when cleaved by Cas13a. SHERLOCK can be adapted to detect DNA by first converting the target DNA to RNA using a RPA.	1 nucleotides	8 × 10^− 21^ M	2–5 h	Detection and genotyping of bacterial and viral infectious disease agents (SARS-CoV-2, Zika). Finding of antibiotic resistance genes. Detection of cancer-associated mutations from circulating cell-free DNA.

RPA: Recombinase Polymerase Amplification

Overall, SHERLOCK may advance cancer diagnostics, offering a rapid, sensitive, and cost-effective method for detecting genetic mutations and biomarkers associated with cancer. SHERLOCK could become an integral part of personalized medicine, improving early cancer detection, monitoring, and overall patient outcomes.

## Active clinical trials testing therapeutic applications of the CRISPR/Cas12 or CRISPR/Cas13 systems

While several clinical trials testing the efficacy of CRISPR/Cas9 system, in particular immunotherapeutic approaches to different types of advanced tumors, are ongoing (https://clinicaltrials.gov*)* and recently reported [180], no trials are currently active to determine the potential therapeutic application of Cas12- or Cas13-based CRISPR systems in oncology. Two clinical studies are ongoing testing the use of CRISPR/Cas12. One concerns the treatment of Severe Sickle Cell Disease (SCD) (ID NCT04853576) (Table 4) [139], an inherited blood disorder involving a defective hemoglobin synthesis, due to a single base-pair point mutation in the β-globin gene, which may lead to multi-organ failure and affects millions of people throughout the world [181]; the other is aimed at exploiting CRISPR/Cas12 for treatment Transfusion-Dependent Beta Thalassemia (TDT) (ID NCT05444894), the most serious form of beta-thalassemia caused by mutations in the β-globin gene leading to ineffective erythropoiesis and chronic severe anemia [182]. Only one study is currently active that use CRISPR/Cas13-mediated RNA targeting, and it is focused on the treatment of neovascular Age-Related Macular Degeneration (AMD) (ID NCT06031727) (Table 4) [139], an acquired degeneration of the retina, which affects one in eight people 60 years of age or older and is the most common cause of irreversible blindness in older persons in developed countries [183].

**Table 4:** Active clinical trials testing therapeutic approaches based on the CRISPR/Cas12 or Cas13 systems [139]

NCT Number	Study Title	Study Status	Study Overview	Sponsor	Phase
NCT04853576	A Study Evaluating the Safety and Efficacy of EDIT-301 in Participants With Severe Sickle Cell Disease (RUBY)	RECRUITING	Multicenter study to evaluate the safety and efficacy of a single dose of EDIT-301 in subjects with severe sickle cell disease. *Treatment*: EDIT-301 (autologous gene edited (CD)34^+^ hematopoietic stem cells, by Cas12a) administered as a one-time intravenous infusion, after myeloablative conditioning with busulfan. *Outcome measures*: − Proportion of subjects achieving complete resolution of severe vaso-occlusive events. − Frequency and severity of adverse events.	Editas Medicine	Phase 1/2
NCT05444894	EDIT-301 for Autologous Hematopoietic Stem Cell Transplant (HSCT) in Participants With Transfusion-Dependent Beta Thalassemia (TDT)	RECRUITING	Single-arm, multicenter study evaluating the safety, tolerability, and efficacy of a single unit dose of EDIT-301 in adult participants with TDT, age 18 to 35 years. *Treatment*: EDIT-301 (autologous gene edited (CD)34^+^ hematopoietic stem cells, by Cas12a) administered as a one-time intravenous infusion, after myeloablative conditioning with busulfan. *Outcome measures*: − Proportion of participants achieving engraftment. Frequency and severity of adverse events.	Editas Medicine, Cambridge, MA, USA	Phase 1/2
NCT06031727	CRISPR/Cas13-mediated RNA tarGeting tHerapy for the Treatment of Neovascular Age-related Macular Degeneration Investigator-initiated Trial (SIGHT-I)	RECRUITING	Multicenter trial to evaluate the safety, tolerability, and efficacy of CRISPR/Cas13 RNA-editing therapy HG202, targeting knock-down of Vascular Endothelial Growth Factor A (VEGFA), in the treatment of neovascular Age-related Macular Degeneration (nAMD). *Treatment*: Once unilateral subretinal injection of HG202, a CRISPR/Cas13 RNA-editing therapy, which use one single vector to partially knock-down the expression of VEGFA and thus inhibit choroidal neovascularization formation in patients who are either responsive or non-responsive to anti-VEGF agents. *Outcome measures*: Incidence and severity of ocular and systemic adverse events.	HuidaGene Therapeutics, Shanghai, PRC	Phase 1

## Outstanding questions

### What challenges must be overcome to move Cas12- and Cas13-based CRISPR systems into clinical oncology?

Moving Cas12- and Cas13-based CRISPR systems into clinical oncology presents challenges related to technical, safety, distribution, ethical, regulatory and translational research aspects, that must be overcome to harness their potential for cancer treatment [184]. Some of the key issues to be addressed are as follows.

- **Delivery mechanisms.** Efficient and targeted delivery of Cas proteins and guide RNA to specific cells and tissues is crucial. Delivery methods need to ensure that Cas proteins and guide RNA reach the desired cells and tissues without causing off-target effects. Current delivery vectors, such as viral vectors (e.g. Adeno-Associated Virus, lentivirus) and non-viral methods (e.g. lipid nanoparticles) [185], have limitations, regarding specificity, efficiency, and safety, in ensuring effective systemic delivery of CRISPR components for metastatic cancers that have spread to multiple sites.
- **Off-target effects.** Although Cas13 targets RNA rather than DNA, off-target effects are still a concern. Unintended RNA cleavage can disrupt normal cellular functions and lead to adverse effects. Improving the specificity of Cas13 to minimize off-target activity is essential.
- **Long-term effects and stability.** Understanding the stability and long-term effects on patient health of DNA editing by Cas12, or RNA edits made by Cas13 is crucial for effective clinical applications. Efficacy and long-term safety represent major concerns that remain to be adequately addressed in preclinical studies [186].
- **Immunogenicity**. All the components of CRISPR-based therapy, Cas9, Cas12 and Cas13 proteins, gRNA, viral or non-viral vectors, can elicit adverse immune reactions. Reducing their immunogenicity is essential to ensure the clinical availability and utility of CRISPR therapeutics [187].
- **Patient selection and genetic diversity.** Patients’ genetic diversity can affect the success of both Cas12- and Cas13-based therapies [188, 189]. Personalized approaches may be required to account for variations in genetic backgrounds, which can influence the efficiency and safety of the treatment.

Overcoming these challenges through robust preclinical studies, rigorous clinical trials, multidisciplinary collaboration, and developing specific guidelines and standards for the use of CRISPR, will be crucial to provide opportunities for the successful clinical application of the next-generation CRISPR-based systems in modern precision oncology.

## Data Availability

No datasets were generated or analysed during the current study.

## Abbreviations

AAV: adeno-associated virus
AMD: age-related macular degeneration
CAR-T: chimeric antigen receptor T cell
Cas: CRISPR-associated protein
CRISPR: clustered regularly interspaced short palindromic repeats
crRNA: CRISPR RNA
CHANGE-seq: circularization for high-throughput analysis of nuclease genome-wide effects by sequencing
ctDNA: circulating tumor DNA
dCas: nuclease-deficent Cas
DETECTR: DNA endonuclease targeted CRISPR trans reporter
Digenome-seq: Cas9 nuclease-digested whole genome sequencing
DSB: double-stranded break
dsDNA/RNA: double-stranded DNA/RNA
GOF: gain-of-function
GUIDE-seq: genome-wide unbiased identification of DSBs enabled by sequencing
gRNA: guide RNA
HDR: homology-directed repair
lncRNA: long non-coding RNAs
Indels: insertions-deletions
LOF: loss-of-function
miRNA: microRNA
MDSCs: myeloid-derived suppressor cells
PAM: protospacer adjacent motif
PARPi: poly(ADP-ribose) polymerase inhibitor
PRRs: pattern recognition receptors
RS-1: RAD51-stimulatory compound 1
RuvC: an endonuclease domain named for an *E. coli* protein involved in DNA repair
SaCas9: *Staphylococcus aureus* Cas9
SCD: severe sickle cell disease
sgRNA: single guide RNA
SHERLOCK: specific high sensitivity enzymatic reporter unLOCKing
SpCas9: *Streptococcus pyogenes* Cas9
ssDNA/RNA: single-stranded DNA/RNA
TCR: T cell receptor
tracrRNA: trans-activating crRNA
